# Effect of cough technique and cryogen gas on temperatures achieved during simulated cryotherapy

**DOI:** 10.1186/1472-6874-7-16

**Published:** 2007-10-01

**Authors:** Yancy Seamans, Charlie Loesel, Jose Jeronimo, John Sellors, Philip E Castle

**Affiliations:** 1 PATH, Seattle, WA, USA; 2 Division of Cancer Epidemiology, National Cancer Institute (NCI), National Institutes of Health (NIH), Department of Health and Human Services (DHHS), Bethesda, MD, USA

## Abstract

**Background:**

Cryotherapy is often used to treat cervical precancer in developing countries. There are different methods and cryogen gases used for cryotherapy, including the freeze-flush-freeze (cough) technique employed to minimize gas blockage. However, there is limited information to compare their effectiveness.

**Methods:**

Using a tissue model, we compared temperature-time curves for four cryotherapy methods: uninterrupted freezing with nitrous oxide (N_2_O) and carbon dioxide (CO_2_), and two methods using a standard and extended version of the cough technique with CO_2_.

**Results:**

Uninterrupted freezing with both N_2_O and CO_2 _produced tissue temperatures less than -20°C (-40°C and -30°C respectively). CO_2 _cryotherapy procedures using the two cough techniques produced temperatures greater than -20°C in the model tissue.

**Conclusion:**

CO_2 _cryotherapy using the cough technique may not achieve sufficiently low temperatures to produce the desired therapeutic effect. Other alternatives to the prevention of gas blockage should be developed.

## Background

Cryotherapy is a method for the treatment of cervical precancerous lesions [1,2] that is considered the most suitable option to use in low-resource settings with underserved populations because it is low cost, requires no anaesthesia or electricity, and has a low complication rate [3]. Although nitrous oxide (N_2_O)-based cryotherapy is effective [4,5], its limited availability and higher cost in developing countries make carbon dioxide (CO_2_)-based cryotherapy a more practical choice. The reported limitations to using CO_2 _include blockage of the cryogun during the cryotherapy procedure, inherently warmer tip temperatures due to the physical properties of the gas, and indications of lower therapeutic effect due to insufficient depth of tissue destruction during studies that employed a freeze-flush-freeze cycle ("cough technique") [6].

The "cough technique" has been recommended to avoid cryogun blockage during treatment [3]. It involves a cyclic momentary thawing of the cryogun probe by systematically interrupting freezing. Although the cough technique is currently being used in developing-country low-resource settings, there are no published data on the validity of this approach or the impact of using the cough technique on the depth of tissue necrosis generated by these methods. We developed a system to compare the temperature profile at the probe and in a tissue model of three different CO_2_-based methods (an uninterrupted freeze cycle, the standard cough technique, and an extended cough technique) as well as the well-characterized N_2_O uninterrupted freeze.

## Methods

**Figure 1:**
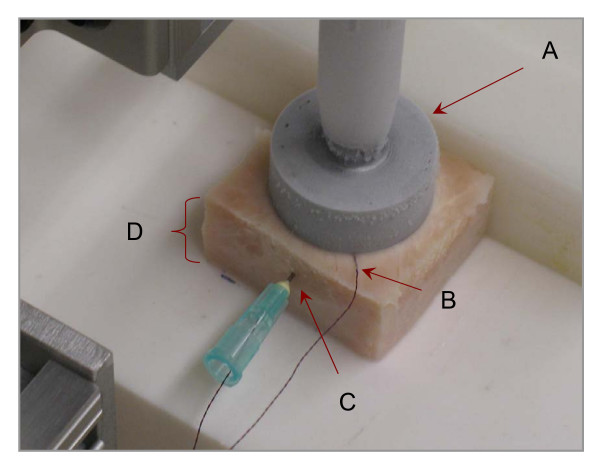
A photograph of the model system used to measure tissue temperature after applying a cryotip (probe) (A). The temperature was measured using thermocouples at the probe (B) and at a depth of 5 mm into the model tissue (C), composed of a uniform slice of turkey (D).

We measured temperatures for a three-minute procedure using the Wallach LL100 cryogun with a T-2500 2.5 cm diameter flat probe (Wallach Surgical Devices – Orange, Connecticut) with thermocouple microprobes IT-23 (Physitemp – Clifton, New Jersey) at the probe/tissue interface and within the tissue sample at a depth of 5 mm. The tissue model used was sliced turkey deli meat (30 mm long × 30 mm wide × 15 mm deep) at room temperature. The probe was applied to the tissue at an approximate pressure of 0.7 kg (6.9 N) by fixing the probe vertically perpendicular to the tissue surface and using the weight of the cryogun to standardize the force. A photograph of the model system is shown in Figure 1.

We evaluated three CO_2_-based cryotherapy techniques: uninterrupted freezing, the standard cough technique, and an extended cough technique. For the standard cough technique, the operator depressed the defrost key every 15 to 20 seconds for approximately one second, released it, and immediately reengaged the freeze key. To employ the extended cough technique, the operator inserted a short pause (approximately onesecond) after releasing the defrost key and before reengaging the freeze key. During this pause, the operator waited to hear the cryogun pulse valve open before reengaging the freeze key.

Individual measurements for each method were reproduced five times. Uninterrupted freezing using N_2_O cryotherapy was our referent standard.

The five runs for each group were averaged for a mean curve. The Kruskal-Wallis test (for nonparametric data) was used to test for statistical differences (P < 0.05) between median temperatures for the mean curve for each cryotherapy technique.

## Results

**Figure 2:**
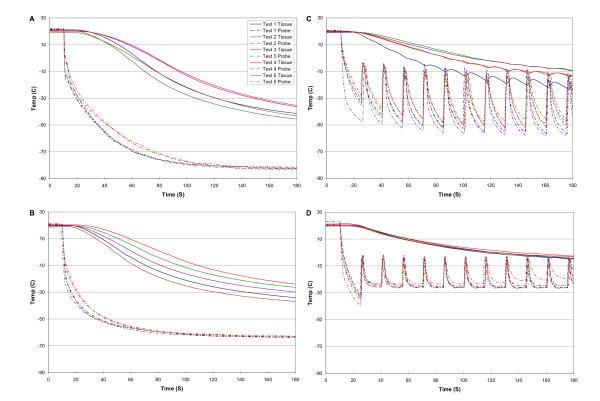
A comparison of temperature-time curves of the probe and model tissue for N_2_O cryogen (A), CO_2 _cryogen (B), CO_2 _cryogen using the extended cough technique (C), and CO_2 _cryogen using the standard cough technique (D).

Figure 2 shows the temperature-time curves (n = 5 for each method) for N_2_O cryotherapy with uninterrupted freezing (A), CO_2 _cryotherapy with uninterrupted freezing (B), with the extended cough technique (C), and with the standard cough technique (D). A summary of the data is shown in Table 1. In our model system, N_2_O cryotherapy with uninterrupted freezing achieved the lowest median temperature at the probe (P = 0.0001) and at a 5 mm depth in the "tissue" (P = 0.0001) compared to other methods. Similarly, N_2_O cryotherapy with uninterrupted freezing was able to achieve the lower median temperature at the probe (P = 0.0001) and at a 5 mm depth in the "tissue" (P = 0.0001) than CO_2 _cryotherapy with uninterrupted freezing. Although the extended cough technique for CO_2 _cryotherapy achieved a lower temperature for the probe (P = 0.0001) and tissue (P = 0.0001) measurements than the standard cough technique, the extended cough technique only achieved a minimum tissue temperature of -14°C (versus -40°C for N_2_O cryotherapy with uninterrupted freezing and -30°C for CO_2 _cryotherapy with uninterrupted freezing).

**Table 1:** A summary of the temperature-time curves from 10 to 180 seconds

		**N**_2_**O**	**CO**_2_	**CO**_2 _**(Extended Cough)**	**CO**_2 _**(Standard Cough)**
**Probe**	Mean (°C)	-69	-56	-34	-21
	Median (°C)	-77	-61	-37	-24
	Minimum (°C)	-82	-63	-53	-35
	Time to -20°C (sec)*	6	4	7	7

**Tissue**	Mean (°C)	-12	-10	1	6
	Median (°C)	-17	-15	-2	3
	Minimum (°C)	-40	-30	-14	-4
	Time to -20°C (sec)*	92	104	n/a	n/a

*Time to -20°C started at 10 seconds.

## Conclusion

This laboratory-based evidence indicates that both cough techniques, used to prevent blockage of the cryogun during CO_2 _cryotherapy, significantly affected the temperature achieved at a 5 mm depth and did not achieve the minimum temperature of -20°C required for tissue destruction and therapeutic effects [7]. When compared to the temperatures achieved with N_2_O cryotherapy, the referent treatment shown to be effective for treatment of cervical precancer, it suggests that neither cough technique is likely to provide the same therapeutic effect as uninterrupted freeze. In addition to the importance of minimum temperature required for necrosis, there are other factors such as cooling rate and total time at the minimum temperature [8] that are also affected by the cough technique.

The standard cough technique was limited in the minimum temperature achieved due to the design of the Wallach cryogun internal pulse valve. The valve must be closed during the defrost cycle and open during the freeze cycle. The rapid cycling of the standard cough technique does not allow time for the valve to reopen following defrost. It remains closed causing the cryogun to function at an intermediate temperature between defrost and freeze. The extended cough (increased time interval between the freeze cycles) allows time for the valve to open, ensuring proper functioning during the subsequent freeze cycle. However, both methods caused significant temperature variation at the cryogun probe and limit the minimum temperature achieved in the tissue.

We acknowledge that this is a model system in which temperature was measured at a specific depth rather than the tissue necrosis, the true desired outcome. Additionally, because the study employed model tissue rather than human tissue, additional human studies will be needed to confirm our laboratory findings. Thus, we can only make qualitative assessments between different methods. While our ability to extrapolate as to what will happen in live tissue is limited, it can be anticipated that these higher than desirable temperatures will be further exacerbated because of the warming effect of the blood supply leading to higher tissue temperatures than observed in our model system.

This evaluation was limited to use of the Wallach LL100; minimum probe and tissue temperatures may vary if other cryoguns are used. In addition, frequency of device blockage is undetermined when using CO_2 _with other cryotherapy devices, which may not incorporate an "active" defrost feature and hence the cough technique may or may not be possible.

Based on these data, technical improvements for CO_2 _cryotherapy are necessary to make it a more useful treatment modality for cervical precancer in underserved populations who cannot get surgical excision. This may include methods to dry or condition CO_2 _to prevent the blockage in the cryogun during the procedure[9].

## Competing interests

The author(s) declare that they have no competing interests.

## Authors' contributions

YS helped to design and coordinate the study and to draft the manuscript. CL carried out the study and helped to draft the manuscript. JJ helped to design the study, perform the statistical analysis, and to draft the manuscript. JS helped to design the study and to draft the manuscript. PEC helped to design the study, perform the statistical analysis, and to draft the manuscript. All authors have read and approved the final manuscript.

## Pre-publication history

The pre-publication history for this paper can be accessed here:


